# Long-term clinical outcomes in patients between the age of 50–70 years receiving biological versus mechanical aortic valve prostheses

**DOI:** 10.1093/ejcts/ezaf033

**Published:** 2025-02-01

**Authors:** Jeremy Chan, Pradeep Narayan, Daniel P Fudulu, Tim Dong, Hunaid A Vohra, Gianni D Angelini

**Affiliations:** Department of Cardiac Surgery, Bristol Heart Institute, University of Bristol, Bristol, UK; Department of Cardiac Surgery, Rabindranath Tagore International Institute of Cardiac Sciences, Narayana Health, India; Department of Cardiac Surgery, Bristol Heart Institute, University of Bristol, Bristol, UK; Department of Cardiac Surgery, Bristol Heart Institute, University of Bristol, Bristol, UK; Department of Cardiac Surgery, Bristol Heart Institute, University of Bristol, Bristol, UK; Department of Cardiac Surgery, Bristol Heart Institute, University of Bristol, Bristol, UK

**Keywords:** Aortic valve replacement, Mechanical prosthesis, Biological prosthesis, Transcatheter aortic valve replacement

## Abstract

**OBJECTIVES:**

The last 2 decades have seen an incremental use of biological over mechanical prostheses. However, while short-term clinical outcomes are largely equivalent, there is still controversy about long-term outcomes.

**METHODS:**

All patients between the ages of 50 and 70 years undergoing elective/urgent isolated aortic valve replacement at our institute between 1996 and 2023 were included. Trends, early, and long-term outcomes were investigated.

**RESULTS:**

A total of 1708 (61% male) patients with a median age of 63.60 (interquartile range: 58.28–67.0) years were included of which 1191 (69.7%) received a biological prosthesis. After inverse propensity score weighting, there were no short-term differences when comparing patients receiving biological and mechanical valves. However, patients who received mechanical prostheses had better long-term survival (*P* < 0.001). Sub-group analysis revealed that patients with biological size 19 mm prosthesis had the worst long-term survival. Patients with a size 21-mm mechanical prosthesis had better survival compared to both size 19-mm [hazard ratio (HR) 0.25, 95% confidence interval (CI) 0.17–0.37, *P* < 0.001], 21-mm (HR 0.33, 95% CI 0.23–0.48, *P* < 0.001) and 23-mm (HR 0.40, 95% CI 0.27–0.60, *P* < 0.001) biological prosthesis. Additionally, patients with severe patient–prosthesis mismatch exhibited the lowest survival rate compared to those with moderate or no (HR 1.56, 95% CI 1.21–2.00, *P* < 0.001).

**CONCLUSIONS:**

Patients aged between 50 and 70 years with a mechanical aortic prosthesis had better long-term survival compared to those with a biological prosthesis. Our study underscores the need for a critical re-evaluation of prosthesis selection strategies in this age group.

## GRAPHICAL ABSTRACT

**Figure:**
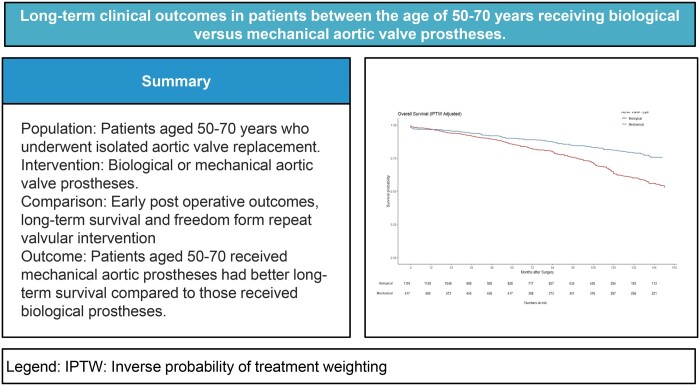

## INTRODUCTION

Existing guidelines advocate for the use of mechanical valves in patients below the age of 50, while favouring biological valves for those above the age of 65 or 70 [1, 2]. The guidelines leave the choice to the discretion of surgeons and patients between 50 and 70 years, acknowledging the unique considerations and preferences inherent to the decision-making process. A global surge in the adoption of biological aortic valve prostheses among patients aged between 50 and 70 years has been observed. This is primarily attributed to the widespread adoption of transcatheter techniques and the inherent appeal for avoiding long-term anticoagulation while undergoing surgical aortic valve replacement (SAVR) [3–6].

Despite documented short-term clinical outcomes being comparable between mechanical and biological prostheses in this age group, considerable controversy surrounds the long-term impact on survival and the likelihood of repeat valvular interventions [7–10]. Apart from structural valve degeneration, patient–prosthesis mismatch (PPM) is an important cause of nonstructural valve deterioration that can also influence re-interventions and long-term survival [11, 12].

Our study endeavours to contribute valuable insights into the dynamics of aortic valve prosthesis selection within the 50–70 age demographic. It also examines trends, reports early outcomes and long-term survival rates, the incidence of repeat valve interventions and provides critical insights into PPM, in patients receiving biological or mechanical aortic valve prostheses at our institution over a 27-year period.

## METHODS

All consecutive patients aged between 50 and 70 years who underwent isolated SAVR from to 1996–2023 (first quarter in 2023 only) at the Bristol Heart Institute were included in this study. Exclusion criteria included acute/chronic infective endocarditis, emergency/salvage procedures, previous cardiac surgery and recipients of an allograft/homograft. Based on the type of prosthesis used, the patients were placed into 2 groups (biological or mechanical). The primary objective was to compare long-term survival among patients between 50 and 70 years of age receiving mechanical or biological valves after inverse probability of treatment weighting (IPTW) analysis. The secondary objective was to assess trends, early clinical outcomes and repeat valvular intervention. In addition, the effect of valve size and PPM on long-term outcomes.

This study was conducted following the principles of the Declaration of Helsinki. The local audit committee approved the study and waived the requirement for individual patient consent (Project code: CARDS/SE/2024-25/01).

### Early clinical outcomes and long-term survival

Early clinical outcomes were defined as events occurring within the hospital or 30-day postsurgery period, obtained from prospectively collected data. For long-term survival analysis, patient details were cross-referenced with the death registry of the Office for National Statistics alongside our clinical database (Patient Analysis & Tracking System, Bristol, UK) using the individual patients’ National Health Service number. Survival and follow-up data were available up to the 30 October 2023. Repeat valvular intervention was defined as the occurrence of either a repeat SAVR or a valve-in-valve transcatheter aortic valve implantation (TAVI). Long-term survival and repeat valvular intervention events were represented as time-to-event data through Kaplan–Meier plots.

### Valve size and PPM

The impact of implanted aortic valve prosthesis size on long-term survival was investigated. The influence of PPM on long-term survival was evaluated by dividing patients into 3 groups: those without, with moderate and with severe PPM. This classification was based on the published effective orifice area (EOA) data specific to each model and size as previously described by Dismorr *et al.* and the imaging assessment of prosthetic heart valves endorsed by 4 Societies of Echocardiography/Cardiovascular imaging [13, 14]. The body mass index (BMI) (kg/m^2^) was defined as: (weight, kg)/(height, m)2 and the body surface area (m^2^) was calculated using the Mosteller formula as defined as [(height, cm) × (weight, kg)/3600]^1/2^. Lastly, the index EOA (iEOA) was calculated using the following formula: EOA/body surface area^2^. The Valve Academic Research Consortium 3 definition where no PPM defined as iEOA > 0.85 cm^2^ in patients with BMI < 30 kg/m^2^ or iEOA > 0.70 cm^2^ in patients with BMI ≥ 30 kg/m^2^, moderate PPM as iEOA 0.85–0.66 cm^2^ in patients with BMI < 30 kg/m^2^ or iEOA 0.70–0.56 cm^2^ in patients with BMI ≥ 30 kg/m^2^ and severe PPM as iEOA ≤ 0.65 cm^2^ in patients with BMI < 30 kg/m^2^ or iEOA ≤ 0.55 cm^2^ in patients with BMI ≥ 30 kg/m^2^ [15]. The adjacent-size EOA was used in models where the smallest or largest size was not known.

### Statistical analysis

Continuous variables were reported as median and interquartile range (IQR). Categorical variables were reported as frequencies and percentages. Pearson’s chi-squared test, Wilcoxon rank-sum test and one-way/multifactor analysis of variance were used to compare 2 categorical variables, for comparison between means of 2 continuous, independent samples and to compare between 3 continuous variables, respectively. After inverse propensity score matching, McNemar's Chi-squared test was used to compare categorical variables.

Survival curves were expressed as Kaplan–Meier plots to plot time-to-event end-points with the *x*-axis being months after surgery against the *y*-axis as overall survival/event probability. The numbers at risk are expressed as a risk table below the *x*-axis in each individual survival curve. Log-rank analysis was used to compare between-group significance. When there were 3 or more groups for comparison, the Cox regression model was used to quantify the effect size by including more than one variable in a regression model to account for the effects of multiple variables (3 in our case). The results were expressed as hazard ratios and confidence intervals of the hazard ratios.

IPTW was performed to create 2 balance groups before direct comparison. The effectiveness of IPTW in balancing covariates across 2 classes was previously described [16]. In addition, the advantages of IPTW over propensity score matching, in studies with a small number of events and/or a large number of confounders were shown by Chesnaye *et al.* [17]. The effectiveness of IPTW before and after was evaluated using the standardized mean difference (SMD) between groups. An SMD of <0.1 is considered to be adequate. Supplementary Material, Fig. S1 showed the SMD of preoperative characteristics before and after IPTW.

Binary logistic regression was performed using the baseline patient demographics and comorbidities to predict factors associated with the use of mechanical aortic valve in the whole cohort. In addition, multiple linear regression was used to predict the long-term survival with pre-, intra-, post-operative characteristics in Supplementary Material, Tables S1 and S2. Generalized variance inflation factors (GVIFs) were calculated to assess multicollinearity in the regression models. To account for differences in the degrees of freedom among variables, we used the adjusted measure with the formula of GVIF^(1/(2 * Df)). Adjusted GVIFs values <2 indicate low multicollinearity, which was observed for all variables in our models (Supplementary Material, Fig. S3). R (version 4.2.3, R Foundation for Statistical Computing, Vienna, Austria) and R Studio (version 1.4.1103, RStudio, PBC) were used to perform statistical analysis. Graphs and tables were created using R (version 4.2.3, R Foundation for Statistical Computing, Vienna, Austria) and Microsoft Office 365 (version 16.0.14026, Microsoft Corporation, Washington, USA).

## RESULTS

### Patient characteristics, short- and long-term clinical outcomes in the whole cohort

A total of 1708 (61% male) patients with a median age of 63.6 (IQR 58.28–67.0) years underwent isolated SAVR at our institution during the study period. The median follow-up was 98 months (IQR 56,134, range: 0–284). Seventy-five percent of the procedures were performed in an elective setting. Of the 1191(69.73%) patients who received a biological prosthesis, 82% (*n* = 932) received an Edwards Perimount or Magna aortic valve, followed by the 9% (*n* = 96) of Trifecta aortic valve. The most commonly used mechanical prosthesis was Sorin Bicarbon (72%, *n* = 370), followed by St Jude mechanical (16%, *n* = 82) (Supplementary Material, Fig. S2). The primary valvular pathology was aortic stenosis (76.63%), followed by mixed (12.19%) and regurgitation (11.18%).

**Figure 1:**
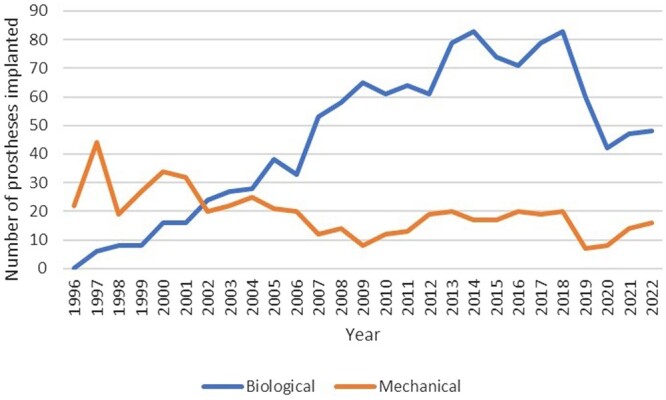
The trend in the number of biological and mechanical aortic valve prosthesis used from 1996 to 2022.

Since 1996, the prevalence of biological prostheses has steadily risen, and in 2002, it surpassed the utilization of mechanical prostheses. During the coronarvius disease (COVID-19) period, the same trend was maintained but the total number of valve implants was reduced (Fig. 1).

Patients who received a mechanical prosthesis when compared to those receiving a biological prosthesis were younger [median 60.50 (IQR 55.4–65.1) vs 64.8 (IQR 60.0–68.0), *P* < 0.001], had a lower incidence of diabetes (9.09% vs 15.2%, *P* = 0.003) and less likely to had previous percutaneous coronary intervention (PCI) (0.97% vs 3.02%, *P* = 0.04) (Supplementary Material, Table S1). There were no clinical differences in early outcomes between patients who received biological or mechanical prostheses (Supplementary Material, Table S2). Completeness of follow-up (long-term survival and repeat valvular intervention, see method for full details) was achieved in 100% of patients.

**Figure 2:**
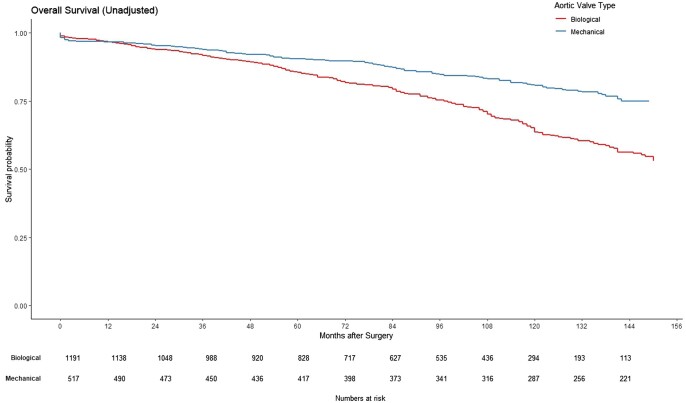
Survival in patients who received biological or mechanical aortic valve prosthesis in the whole cohort before propensity score matching.

Patients who received mechanical prostheses exhibited better long-term survival than those with biological prostheses (log-rank, *P* < 0.001; Fig. 2), with no difference in repeat valvular intervention (log-rank, *P* = 0.053).

### Patient characteristics, short- and long-term clinical outcomes after IPTW

When comparing patients receiving biological and mechanical valve, there were no differences in cardiopulmonary bypass time [median 91.0 (IQR 76.0–106.0) vs 90.0 (IQR 75.0–104.0) min, *P* = 0.41], aortic cross-clamp time [median 67 (IQR 57.0–80.0) vs 66.25 (IQR 56.0–77.03) min, *P* = 0.36], in-hospital mortality (1.28% vs 0.87%, *P* = 0.28), return to theatre (4.74% vs 4.9%, *P* = 0.05), neurological events (3.81% vs 1.92%, *P* = 0.15), post-operative need for dialysis (1.01% vs 0.85%, *P* = 0.86) and deep sternal wound infection at discharge (0.76% vs 0%, *P* = 0.08) (Supplementary Material, Table S2).

**Figure 3:**
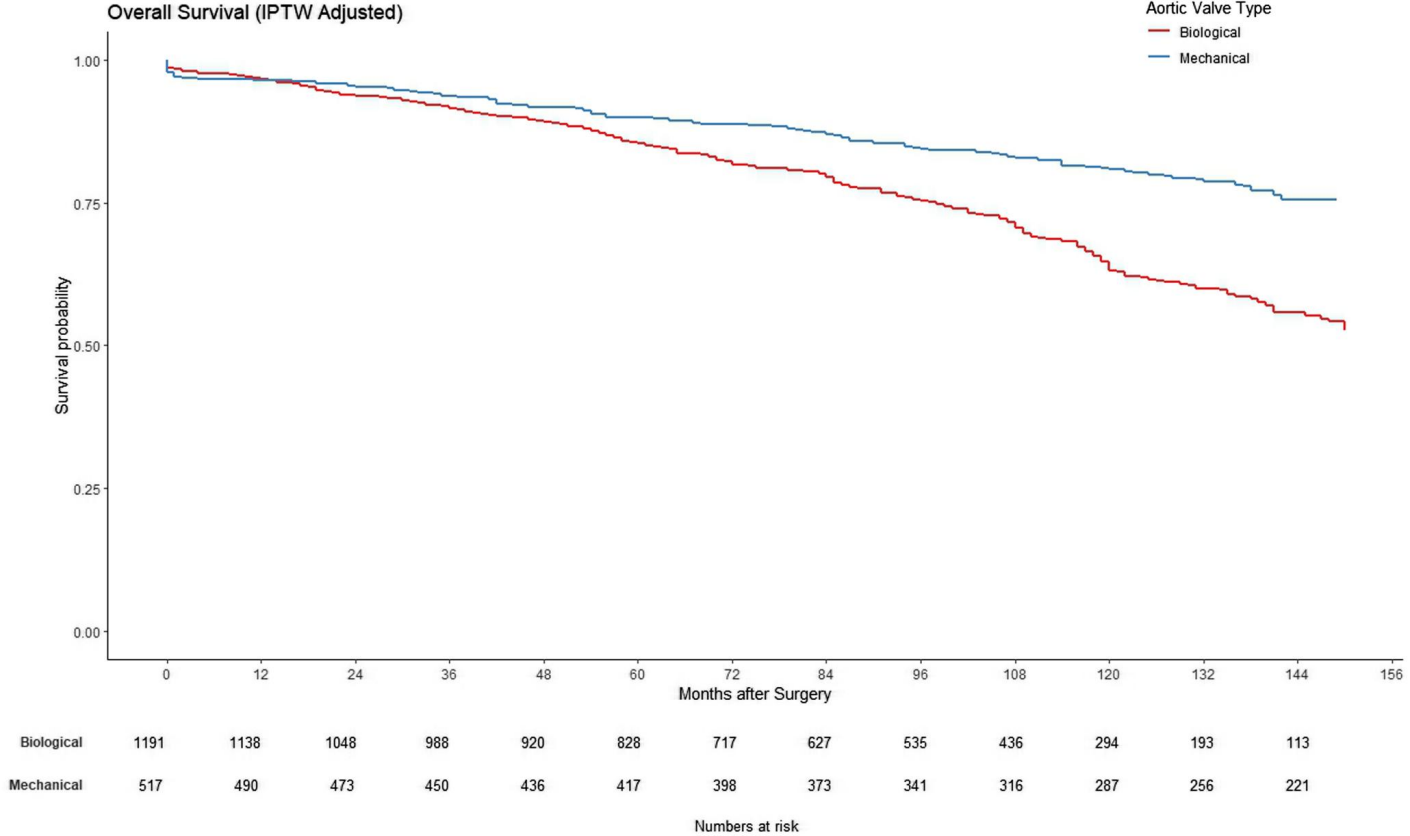
The long-term survival in patient’s age between 50 and 70 years receiving biological or mechanical aortic valve prostheses in the propensity score matching cohort (0: biological, 1: mechanical). IPTW: inverse probability of treatment weighting.

**Figure 4:**
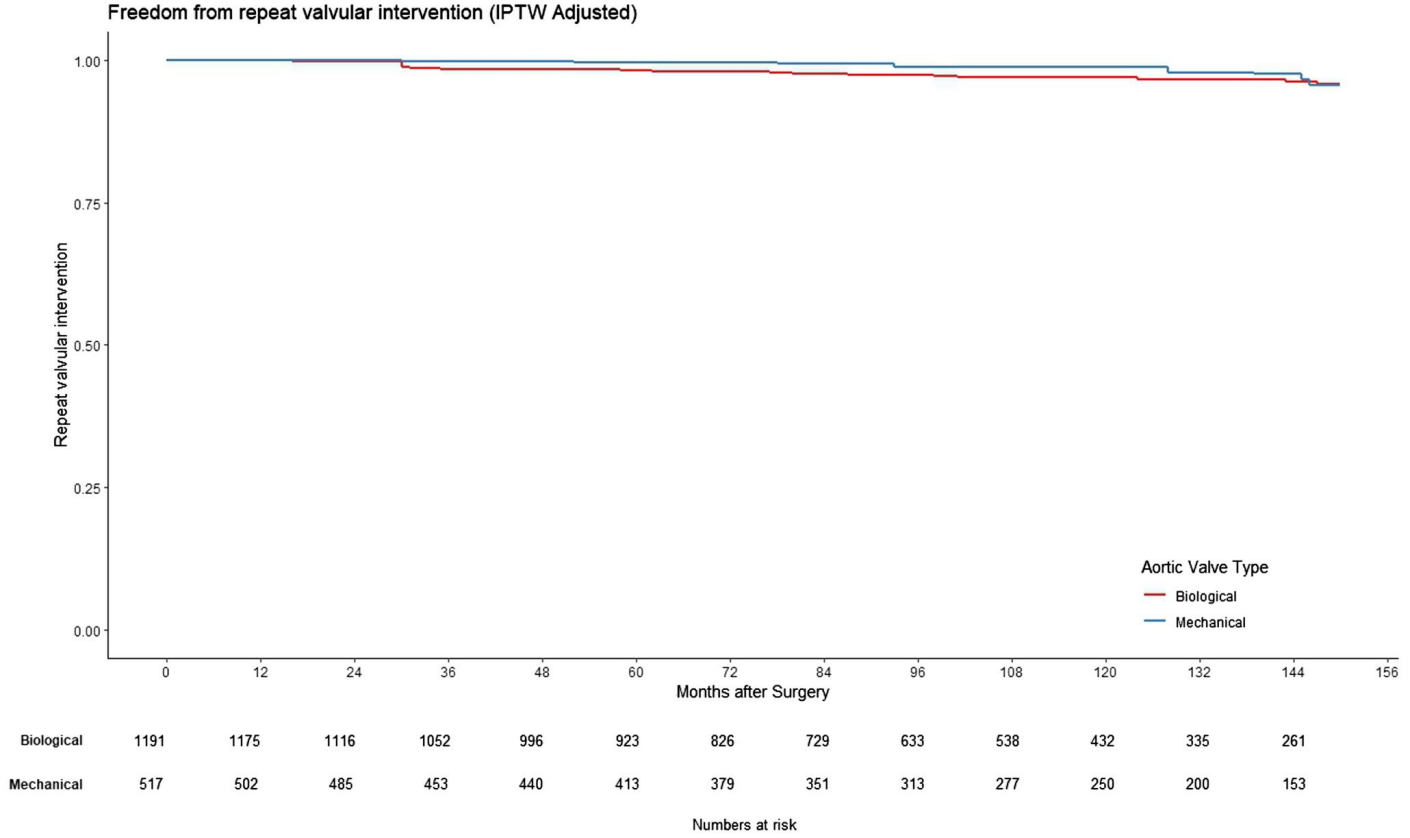
The freedom from repeat valvular intervention in patients age between 50 and 70 years receiving biological or mechanical aortic valve prostheses in the propensity score matching cohort (0: biological, 1: mechanical). IPTW: inverse probability of treatment weighting.

Patients who received mechanical prostheses had better long-term survival than those with biological prostheses (log-rank, *P* < 0.001; Fig. 3). However, there was no significant difference between the groups in the freedom from repeat valvular intervention (log-rank, *P* = 0.51, Fig. 4). Additional analysis comparing perimount versus mechanical valves and excluding Trifecta prosthesis (no longer available) did not change results.

### Valve size and long-term clinical outcomes

**Figure 5:**
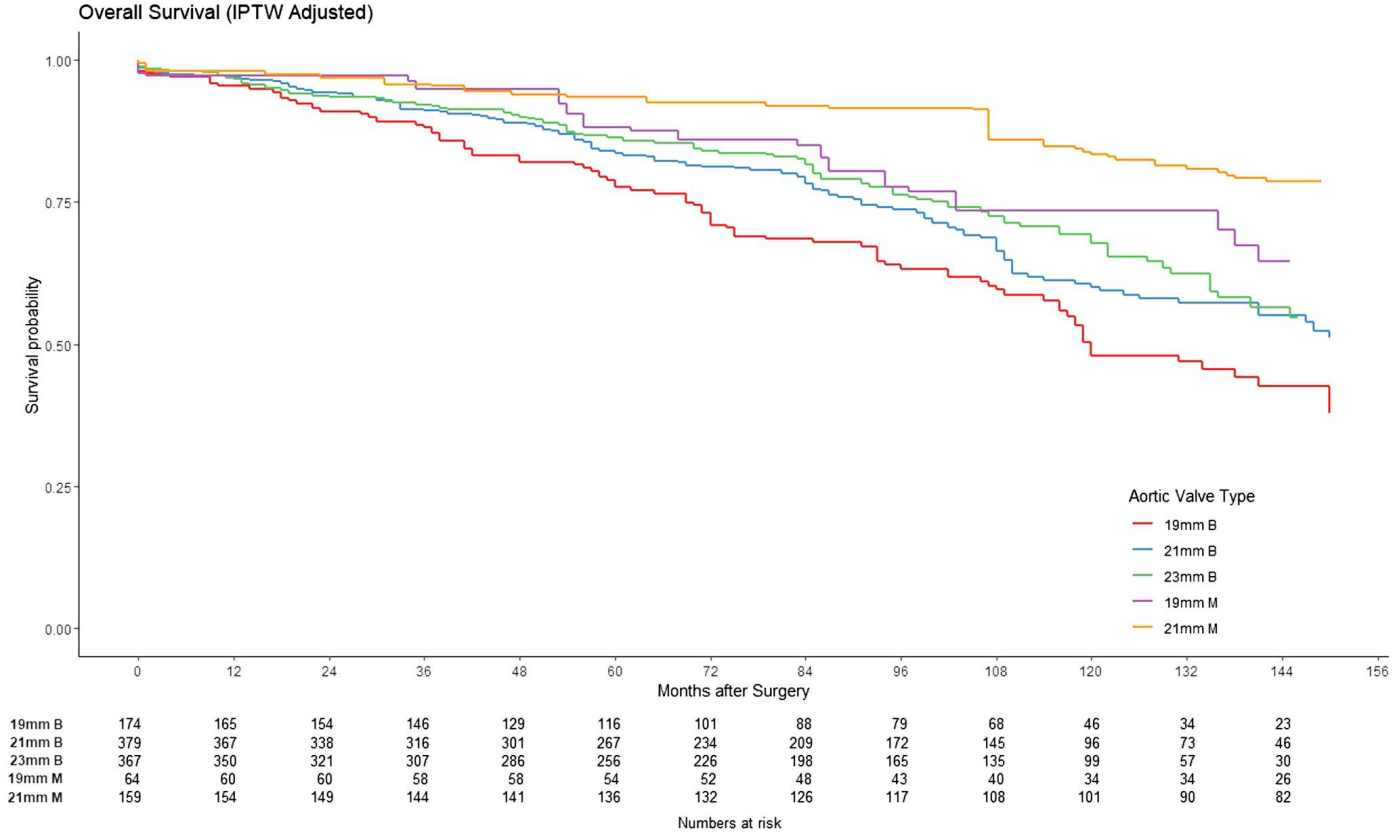
The Kaplan–Meier curve in patients age between 50 and 70 years receiving aortic valve size 19, 21 and 23 mm after propensity score matching, subclassified by prosthesis type (0: 19-mm biological, 1: 21-mm biological, 2: 23-mm biological, 3: 19-mm mechanical, 4: 21-mm mechanical). IPTW: inverse probability of treatment weighting.

In the entire cohort, 174 (14.61%), 379 (31.82%), 367 (30.81%) and 167 (14.02%) patients received biological prostheses sizes 19, 21, 23 and 25 mm, respectively. Mechanical prosthesis sizes 19, 21, 23 and 25 mm were used in 71 (13.73%), 159 (30.75%), 171 (33.08%) and 76 (14.70%) patients, respectively. Long-term survival outcomes for patients receiving prostheses of different sizes in the entire cohort and after PSM are illustrated in (Supplementary Material, Fig. S3, Fig. 5).

After IPTW, patients who received a size 19-mm biological prosthesis had the worst long-term survival among all valve sizes and types. The survival with a size 19-mm biological valve when compared to patients who received a 19-mm mechanical valve was significantly worse (HR 2.11, 95% CI 1.32–3.35, *P* = 0.001). On the other hand, patients who received a size 19-mm mechanical prosthesis demonstrated a better survival compared to 21-mm (HR 0.61, 95% CI 0.40–0.96, *P* = 0.03) biological valves and an equivalent survival when compared with 23 mm (HR 0.75, 95% CI 0.47–1.18, *P* = 0.2) biological prosthesis.

Patients who received a size 21-mm mechanical prosthesis were associated with better long-term survival when compared to patients receiving a biological size 19 mm (HR 0.25, 95% CI 0.17–0.37, *P* < 0.001), 21 mm (HR 0.33, 95% CI 0.23–0.48, *P* < 0.001) and 23 mm (HR 0.40, 95% CI 0.27–0.60, *P* < 0.001). There was no significant difference in the incidence of repeat valvular intervention, regardless of the size and type of prosthesis implanted.

### Patient–prosthesis mismatch

**Figure 6:**
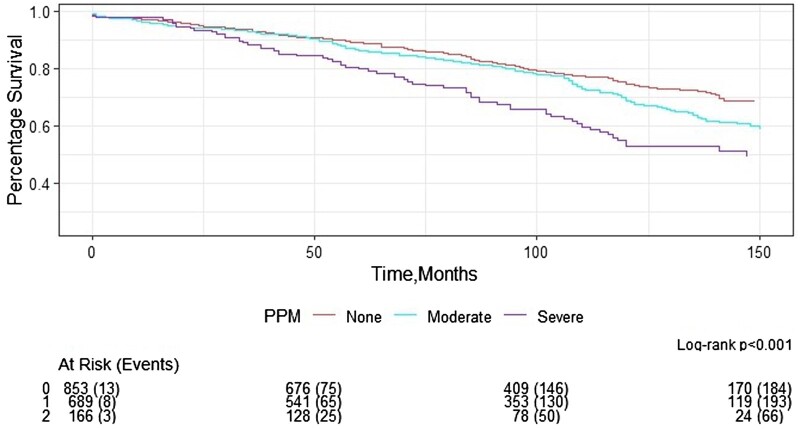
The Kaplan–Meier curve for patients with no, moderate, and severe prosthesis mismatch after surgical aortic valve replacement in the whole cohort (0: no, 1: moderate, 2: severe prosthesis mismatch). PPM: patient–prosthesis mismatch.

In the whole cohort, 853 (49.94%) patients experienced no PPM, while 689 (40.34%) and 166 (9.72%) had moderate and severe PPM, respectively. Patients with severe PPM had the lowest survival rate when compared to those with moderate PPM and no PPM (HR 1.56, 95% CI 1.21–2.0, *P* < 0.001, Fig. 6). Survival benefit remained when comparing patients with no and moderate PPM (HR 0.77, 95% CI 0.64–0.92, *P* = 0.004).

Similar results were also observed in patients who received a biological prosthesis (HR 1.51, 95% CI 1.13–2.02, *P* = 0.005) but not in those who received a mechanical prosthesis (HR 1.40, 95% CI 0.82–2.39, *P* = 0.21).

### Factors predicting the use of mechanical aortic valve and long-term survival

The utilization of mechanical prosthesis was more prevalent in younger patients (OR 0.9, 95% CI 0.88–0.92, *P* < 0.001) and in those with preoperative atrial fibrillation (OR 0.26, 95% CI 0.08–0.89, *P* = 0.031). On the other hand, patients with a more severe canadian cardiovascular society angina classification status (CCS) were more likely to receive a biological prosthesis (OR 1.18, 95% CI 1.07–1.30, *P* < 0.001).

Multiple regression analysis revealed that patients who received a mechanical prosthesis were associated with better long-term survival (*P* < 0.001). Several factors, including weight (*P* = 0.005), preoperative poor left ventricular function (<20%) (*P* = 0.04), urgent operation (*P* = 0.002), extra-cardiac arteriopathy (*P* = 0.039), history of poor mobility (*P* = 0.003), return to theatre (*P* = 0.048) and post-operative dialysis (*P* = 0.007) were associated with worse long-term survival (Supplementary Material, Table S3).

## DISCUSSION

The main findings of our study are that patients receiving mechanical aortic prostheses demonstrated superior long-term survival. This benefit is especially apparent in smaller valve sizes with a 19-mm mechanical valve providing better long-term survival than both 19- and 21-mm bioprosthetic valves, and equivalent to 23-mm bioprosthetic valves. In addition, our study confirms that severe PPM is a significant risk factor for poor long-term survival.

In the UK, there has been a notable shift in aortic valve prosthesis preferences, with approximately 80% of patients aged 60–69 years opting for a biological prosthesis in 2018, a substantial increase from 25% in 1996 [3]. The avoidance of anticoagulation serves as a significant motivator in choosing bioprosthetic over mechanical valves. Furthermore, the evolving landscape of transcatheter techniques globally has contributed to the widespread adoption. The approval by the Food and Drug Administration for the ‘valve-in-valve’ procedure in 2015, specifically for failed surgical bioprosthetic valves, has further expanded the paradigm of lifetime management for aortic valve disease [18, 19]. Despite potential risks such as acute coronary obstruction, coronary sinus sequestration and elevated gradients during valve-in-valve -TAVI, the utilization of TAVI is unabated [20]. While TAVI and ViV-TAVI stand as viable options for the elderly population, their application in younger age groups is a subject of contention.

In the short term, there does not appear to be much difference following SAVR with bioprosthetic or mechanical valves in the age group of 50–70 years. The only randomized controlled trial comparing mechanical and biological valves in the age 55–70 reported no difference in 30-day mortality [21]. Although short-term outcomes hold significance, it is imperative to scrutinize the evidence comprehensively over a longer term. Conflicting findings exist in the literature on survival outcomes in the longer term following SAVR with bioprosthetic or mechanical valves in the age group of 50–70 years. The AUTHEARTVISIT study demonstrated significantly superior survival with mechanical aortic valve prostheses, specifically in patients aged 50–65 years [22]. Another retrospective study affirmed a survival advantage associated with mechanical valves in patients spanning the age range of 50–70 years [23]. In contrast, several studies have reported no survival benefit of mechanical over bioprosthetic valves within this age cohort [9, 21, 24]. Several factors including study design, sample size and the duration and completeness of follow-up perhaps influence this variability. The study design had been varied and included a randomized controlled trial [21], retrospective study [23], PSM studies [9], multicentric [25] and nationwide registry data [8, 22, 24]. Sample sizes have varied between 310 [21] and over 4500 [8] patients. It is worth noting that apart from one study [9], studies with larger sample sizes demonstrated a survival advantage for mechanical valves [8, 22]. The follow-up period has also varied greatly between the studies and has ranged from 5 to 15 years [8, 9, 22, 24]. The mechanism and completeness of follow-up also influence the reported outcomes. As in our study when patient-level record linkage was established with national health-data registers, a more comprehensive and accurate follow-up of outcomes could be acquired, which demonstrated a superior survival for mechanical valves [8, 22].

Risk of reintervention has been reported more commonly with bioprosthetic valves [8, 9, 21, 22]. There are at least 2 studies that have indicated comparable reintervention rates between bioprosthetic and mechanical valves. However, it is important to note that both these studies had relatively short follow-up periods, with the mean follow-up of only 5 [24] and 6.3 years [23]. In contrast, studies with longer follow-up periods consistently show an increased risk of reintervention with bioprosthetic valves [8, 9, 22]. For instance, the AUTHEARTVISIT study reported a hazard ratio of 3.5 for reintervention in patients aged 50–65 undergoing SAVR with bioprosthetic valves [22]. These findings underscore the importance of considering follow-up duration when assessing the risk of reintervention associated with different valve types.

Another important feature of our study is the analysis of outcomes based on valve sizes. In patients with small annulus, a mechanical prosthesis offered superior long-term survival than a biological valve. The survival benefit with a mechanical 19-mm valve prosthesis was in fact superior to not only a similar-sized (19 mm) bio-prosthesis but also a size higher (21 mm). Equivalent survival of a size 19-mm mechanical valve was noted compared to a biological valve 2 sizes higher (23 mm). Based on our study it could be argued that since a 19-mm mechanical valve could provide similar long-term survival benefit compared to a 23-mm bioprosthetic valve, the risk associated with root enlargement and implanting a larger-sized valve could be avoided [26–28].

The reduced survival with 19- and 21-mm bioprosthetic valves seen in our study is likely secondary to PPM and the resultant effects. PPM occurs when the EOA is too small for patients’ haemodynamic requirements. There are 2 methods to define PPM commonly used in the literature, predicted and measured PPM. Like many other reports, we have adopted the predicted PPM method as defined in the Method section. Measured PPM, on the other hand, uses post-operative echocardiography to derive EOA. Our study population with a median BMI of 27 represents the Western population, and the incidence of moderate and severe PPM observed in our series was 40.34% and 9.72%, respectively. The incidence of severe PPM was higher in our institute (9.72%) than in a sub-study from the PARTNER trials (2.1%) using predicted PPM [29]. Thourani *et al.* performed a sub-study of the PARTNER trials, comparing incidences of PPM using predicted and measured PPM. Measured PPM seems to have overestimated the incidence of PPM, and surgeons should be aware of this in their clinical practice as this method is often utilized in TAVI reports. This may give a false impression that SAVR is associated with a significantly higher incidence of PPM when compared with TAVI. Nevertheless, both our and Thourani’s data suggested that severe (predicted) PPM is associated with poorer short-term outcome [29]. In addition, severe PPM was a significant risk factor for poor long-term survival in our cohort, and several studies have demonstrated similar results [11, 12, 30]. The increased gradient may potentially impede left ventricular mass regression and subsequently predispose to faster degeneration of bioprosthetic valves [12, 31, 32]. Left ventricular mass regression has been suggested to be predictive of improved long-term survival [33]. Therefore, surgical techniques to minimize PPM should be considered in this patient’s sub-group.

### Limitation

Our study is subject to several inherent limitations. Firstly, its single-institution design, retrospective nature of prospectively collected data, and absence of randomization render the study susceptible to various forms of bias. The lack of echocardiographic information could potentially have underestimated the true occurrence of structural valve failure. In terms of repeat valvular interventions, only patients who underwent re-do SAVR or valve-in-valve TAVI at our institution were included. We are a supra-regional centre, hence is very unlikely that many patients may have undergone reintervention in other institutions. The cause of death (cardiovascular/noncardiovascular) was not available which is another limitation.

Despite the application of IPTW to mitigate observed imbalances, the potential for residual bias persists due to the inability of IPTW to account for unmeasured confounders such as frailty. The use of biological or mechanical prostheses could potentially have been influenced by individual surgeon’s preferences. Finally, our single-centre study is a limitation and randomized controlled trials remain the gold standard in the evidence hierarchy. Notwithstanding these challenges, we assert that the insights presented in this article add substantive value within the contemporary dialogue surrounding valve selection in the 50–70 age demographic.

## CONCLUSION

Our study has implications for decision-making in SAVR for individuals aged 50–70 years. The evidence supporting superior long-term survival in patients receiving mechanical aortic prostheses urges a reconsideration of the prevailing trend favouring biological valves in this age bracket. The survival benefit is especially apparent in smaller-sized valves.

Our study suggests a need for a thoughtful evaluation of the longevity benefits associated with mechanical valves, especially in smaller sizes, despite the attraction of avoiding long-term anticoagulation with biological valves.

## Supplementary Material

ezaf033_Supplementary_Data

## Data Availability

The data underlying this article will be shared on reasonable request to the corresponding author.

## ABBREVIATIONS

BMI: Body mass index
EOA: Effective orifice area
GVIF: Generalized variance inflation factors
IPTW: Inverse probability of treatment weighting
IQR: Interquartile range
PPM: Patient–prosthesis mismatch
SAVR: Surgical aortic valve replacement
SMD: Standardized mean difference
TAVI: Transcatheter aortic valve implantation
