# Supplementary material for: Long-term clinical outcomes in patients between the age of 50–70 years receiving biological versus mechanical aortic valve prostheses
Source: Eur J Cardiothorac Surg. 2025 Feb 1;67(2):ezaf033. doi: 10.1093/ejcts/ezaf033 (PMC11821269; doi:10.1093/ejcts/ezaf033)
Supplement: ezaf033_Supplementary_Data [file ezaf033_supplementary_data.docx]

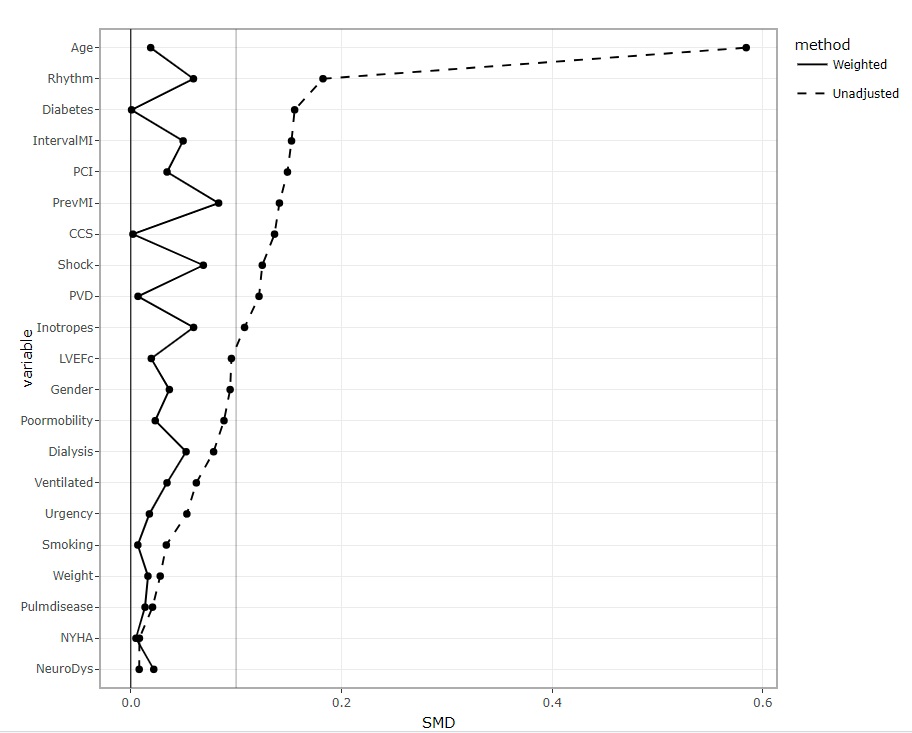


**Figure S1** shows the standardised mean difference (SMD) of the pre-operative variables before and after inverse probability of treatment weighting. (X-axis: SMD, Y-axis: Pre-operative variables)

**Figure S2 showing the biological and mechanical aortic prosthesis implanted in patients age 50-70 years at our institution from 1996-2023**

**
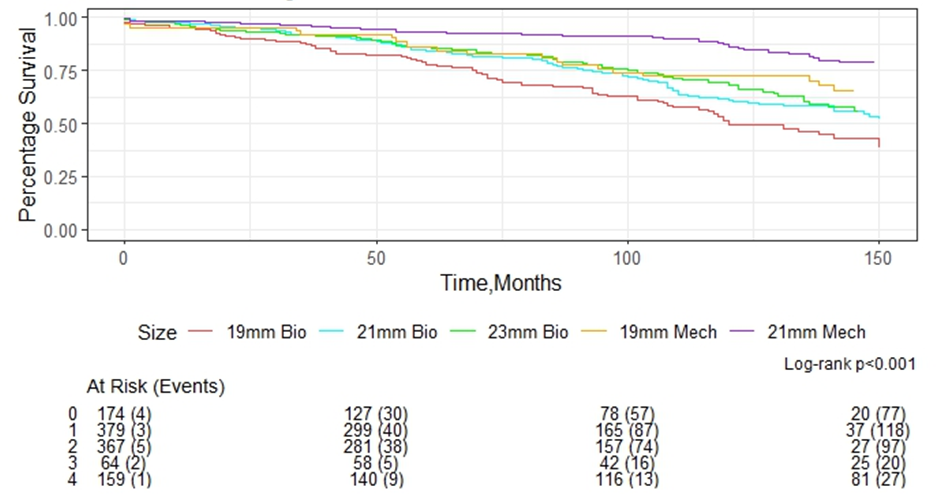
**

Figure S3 shows the Kaplan-Meier curve in patients age between 50-70 years receiving aortic valve size 19, 21, and 23 mm in the whole cohort before IPTW, subclassified by prosthesis type.

| Pre-operative characteristics | Whole Cohort Pre-IPTW | | | Post IPTW | | | |
| --- | --- | --- | --- | --- | --- | --- | --- |
|  | Biological AV (n = 1,191) | Mechanical AV  (n = 517) | p-value | Biological AV (Weight: 1705.2) | Mechanical AV (Weight 1688.8) | SMD | p-value |
| Age (median [IQR]) | 64.8 [60.0, 68.0] | 60.5 [55.4, 65.1] | <0.001 | 63.7 [58.3, 67.0] | 63.3 [58.0, 67.0] | 0.02 | 0.67 |
| Gender (Female) | 476 (39.97%) | 183 (35.40%) | 0.08 | 665.4 (39.02%) | 689.3 (40.82%) | 0.04 | 0.53 |
| Weight (kg)(median [IQR] | 79.0 [70.0, 91.0] | 76.4 [69.3, 89.6] | 0.23 | 79.0 [70.0, 90.9] | 77.0 [69.0, 90.0] | 0.48 | 0.02 |
| ***LVEFC*** |  |  | 0.49 |  |  | 0.99 | 0.02 |
| Good (LVEF > 50%) | 980 (82.28%) | 407 (78.72%) |  | 1383.83 (81.15%) | 1364.40 (80.79%) |  |  |
| Moderate (LVEF 31 - 50%) | 156 (13.10%) | 79 (15.28%) |  | 235.35 (13.80%) | 242.31 (14.35%) |  |  |
| Poor (LVEF 21 - 30%) | 39 ( 3.27%) | 23 ( 4.45%) |  | 63.36 ( 3.72%) | 61.66 ( 3.65%) |  |  |
| Very Poor (LVEF <21%) | 13 ( 1.09%) | 6 ( 1.16%) |  | 17.59 ( 1.03%) | 15.45 ( 0.91%) |  |  |
| ***Urgency*** |  |  | 0.34 |  |  | 0.76 | 0.02 |
| Elective | 903 (75.82%) | 380 (73.50%) |  | 1288.5 (75.55%) | 1262.8 (74.78%) |  |  |
| Urgent | 288 (24.18%) | 137 (26.50%) |  | 416.98 (24.45%) | 425.87 (25.22%) |  |  |
| ***Diabetes*** |  |  | 0.001 |  |  | 0.17 | 0.15 |
| Not Diabetic | 1010 (84.80%) | 470 (90.91%) |  | 1471.49 (86.30%) | 1477.66 (87.50%) |  |  |
| Diet Control | 38 ( 3.19%) | 5 ( 0.97%) |  | 52.80 ( 3.10%) | 17.74 ( 1.05%) |  |  |
| Oral therapy | 111 ( 9.32%) | 32 ( 6.19%) |  | 140.31 ( 8.23%) | 146.14 ( 8.65%) |  |  |
| Insulin therapy | 32 ( 2.69%) | 10 ( 1.93%) |  | 40.59 ( 2.38%) | 47.23 ( 2.80%) |  |  |
| ***Smoking*** |  |  | 0.05 |  |  | 0.02 | 0.16 |
| Never smoked | 526 (44.16%) | 239 (46.23%) |  | 745.83 (43.74%) | 790.79 (46.83%) |  |  |
| Ex smoker | 573 (48.11%) | 216 (41.78%) |  | 825.13 (48.39%) | 705.55 (41.78%) |  |  |
| Current smoker | 92 ( 7.72%) | 62 (11.99%) |  | 134.22 ( 7.87%) | 192.42 (11.39%) |  |  |
| PVD | 54 ( 4.53%) | 12 ( 2.32%) | 0.041 | 65.39 ( 3.83%) | 67.01 ( 3.97%) | 0.92 | 0.007 |
| Preop Rhythm |  |  | 0.016 |  |  | 0.89 | 0.06 |
| Sinus | 1081 (90.76%) | 473 (91.49%) |  | 1547.37 (90.75%) | 1528.69 (90.52%) |  |  |
| AF | 76 ( 6.38%) | 38 ( 7.35%) |  | 119.74 ( 7.02%) | 122.53 ( 7.26%) |  |  |
| Neuro Dys | 22 ( 1.85%) | 9 ( 1.74%) | 1 | 30.98 ( 1.82%) | 35.80 ( 2.12%) | 0.76 | 0.02 |
| CrCl. Category |  |  | 0.73 |  |  | 0.86 | 0.05 |
| normal (CC > 85 ml/min) | 1174 (98.57%) | 508 (98.26%) |  | 1679.37 (98.49%) | 1667.00 (98.71%) |  |  |
| moderate (CC 50-85 ml/m) | 1 ( 0.08%) | 1 ( 0.19%) |  | 1.86 ( 0.11%) | 1.86 ( 0.11%) |  |  |
| severe (CC < 50 ml/min) | 12 ( 1.01%) | 6 ( 1.16%) |  | 17.23 ( 1.01%) | 14.26 ( 0.84%) |  |  |
| CCS Class |  |  | <0.001 |  |  | <0.0001 | 0.33 |
| 0 | 618 (51.89%) | 206 (39.85%) |  | 856.01 (50.20%) | 739.20 (43.77%) |  |  |
| 1 | 147 (12.34%) | 121 (23.40%) |  | 207.01 (12.14%) | 409.59 (24.25%) |  |  |
| 2 | 300 (25.19%) | 130 (25.15%) |  | 442.82 (25.97%) | 368.51 (21.82%) |  |  |
| 3 | 105 ( 8.82%) | 44 ( 8.51%) |  | 163.72 ( 9.60%) | 126.69 ( 7.50%) |  |  |
| 4 | 21 ( 1.76%) | 16 ( 3.09%) |  | 35.62 ( 2.09%) | 44.78 ( 2.65%) |  |  |
| NYHA Class |  |  | 0.15 |  |  | 0.14 | 0.13 |
| 1 | 135 (11.34%) | 74 (14.31%) |  | 193.48 (11.35%) | 245.87 (14.56%) |  |  |
| 2 | 566 (47.52%) | 226 (43.71%) |  | 814.55 (47.77%) | 707.55 (41.90%) |  |  |
| 3 | 432 (36.27%) | 184 (35.59%) |  | 609.83 (35.76%) | 645.87 (38.24%) |  |  |
| 4 | 58 ( 4.87%) | 33 ( 6.38%) |  | 87.33 ( 5.12%) | 89.48 ( 5.30%) |  |  |
| PCI |  |  | 0.03 |  |  | 0.77 | 0.03 |
| No previous PCI | 1155 (96.98%) | 512 (99.03%) |  | 1663.97 (97.58%) | 1649.83 (97.69%) |  |  |
| PCI < 24 hours before surgery | 1 ( 0.08%) | 0 ( 0.00%) |  | 1.00 ( 0.06%) | 0.00 ( 0.00%) |  |  |
| PCI > 24 hours before surgery; same admission | 35 ( 2.94%) | 5 ( 0.97%) |  | 40.22 ( 2.36%) | 38.94 ( 2.31%) |  |  |
| Previous MI |  |  | 0.11 |  |  |  | 0.43 |
| None | 1123 (94.29%) | 501 (96.91%) |  | 3234 (95.30%) | 1620 (95.01%) | 1614(95.59%) |  |
| One | 59 ( 4.95%) | 15 ( 2.90%) |  | 148.29 ( 4.37%) | 75.64 ( 4.44%) | 72.66(4.30%) |  |
| Two or more | 4 ( 0.34%) | 0 ( 0.00%) |  | 4.00 ( 0.12%) | 4.00 ( 0.23%) | 0.00 ( 0.00%) |  |
| Poor Mobility | 21 ( 1.76%) | 4 ( 0.77%) | 0.18 | 24.52 ( 1.44%) | 19.83 ( 1.17%) | 0.75 | 0.02 |
| Ventilated reop | 0 ( 0.00%) | 1 ( 0.19%) | 0.67 | 0.00 ( 0.00%) | 1.00 ( 0.06%) | 0.31 | 0.03 |
| Cardiogenic Shock | 0 ( 0.00%) | 4 ( 0.77%) | 0.01 | 0.00 ( 0.00%) | 4.00 ( 0.24%) | 0.05 | 0.07 |
| Inotropes | 0 ( 0.00%) | 3 ( 0.58%) | 0.05 | 0.00 ( 0.00%) | 3.00 ( 0.18%) | 0.08 | 0.06 |

Table S1. Pre-operative characteristics in patients age between 50-70 years who received biological or mechanical prostheses before and after IPTW (AF: Atrial fibrillation, CCS: Canadian Cardiovascular Society, PCI: Percutaneous Coronary Intervention, NYHA: New York Heart Association, LMS: Left Main stem disease, MI: Myocardial infraction, LVEF: Left ventricular ejection fraction, NeuroDys: Neurological Dysfunction, ES2: Euro Score II, IPTW: Inverse probability treatment weighting, SMD: Standardised mean difference, PVD: Peripheral vascular disease, CrCl.category: Creatinine clearance category, Ventilated Pre op: Require invasive ventilation (including intubation) pre operatively, Inotropes: Inotropic support prior to general anaesthesia.)

| Characteristics | Whole Cohort Pre-IPTW | | | Post IPTW | | |
| --- | --- | --- | --- | --- | --- | --- |
| Variable | Biological AV (n = 1,191) | Mechanical AV (n = 517) | p-value | Biological AV (Weight: 1705) | Mechanical AV (Weight: 1688) | p-value |
| CPB (mins) (Median, IQR) | 91.0 [76.0, 106.0] | 89.0 [75.0, 104.0] | 0.45 | 91.0 [76.0, 106.0] | 90.0 [75.0, 104.0] | 0.41 |
| XClamp time (mins) (Median, IQR) | 67.0 [57.0, 80.0] | 66.0 [56.0, 77.0] | 0.31 | 67.0 [57.0, 80.0] | 66.2 [56.0, 77.0] | 0.36 |
| Mortality | 14 (1.18%) | 5 ( 0.97%) | 0.90 | 21.82 (1.28%) | 14.67 (0.87%) | 0.28 |
| RTT (bleeding, tamponade) | 52 (4.37%) | 21 ( 4.06%) | 0.49 | 80.82 (4.74%) | 82.71 (4.90%) | 0.05 |
| Postop CVA | 18 (2.24%) | 6 (1.44%) | 0.62 | 64.96 (3.81%) | 32.41 (1.92%) | 0.15 |
| Postop Dialysis | 12 (1.01%) | 6 ( 1.16%) | 0.73 | 17.23 (1.01%) | 14.26 (0.85%) | 0.86 |
| Postop DSWI at discharge | 3 (0.86%) | 0 ( 0.00%) | 0.90 | 12.96 (0.76%) | 0 (0.00%) | 0.08 |

Table S2. Intra- and post-operative outcomes in patients aged between 50-70 years who received biological or mechanical prostheses before and after IPTW (CPB: Cardiopulmonary bypass time, RTT: Return to theatre, CVA: Cerebrovascular accident, IPTW: Inverse probability treatment weighting, DSWI: Deep sternal wound infection, XClamp: Aortic Cross clamp).

| **Characteristics** | **OR** | **95% CI** | **p-value** | **Adjusted GVIF value** |
| --- | --- | --- | --- | --- |
| Age at Procedure | 0.28 | -0.42 – 0.97 | 0.437 | 1.06 |
| Gender | -4.52 | -13.89 – 4.84 | 0.343 | 1.28 |
| Weight | -0.34 | -0.58 – -0.10 | 0.005 | 1.15 |
| History of Pulmonary Disease | -1.7 | -13.02 – 9.63 | 0.769 | 1.06 |
| Left ventricular ejection fraction | -7.81 | -18.53 – 2.91 | 0.153 | 1.05 |
| Operative Urgency | -14.21 | -23.11 – -5.32 | 0.002 | 1.08 |
| Diabetes Management | -5.34 | -10.85 – 0.17 | 0.058 | 1.05 |
| Smoking Status | -4.15 | -10.09 – 1.80 | 0.172 | 1.04 |
| Peripheral artery disease | -20.3 | -39.61 – -1.00 | 0.039 | 1.07 |
| Rhythm | -11.84 | -26.38 – 2.69 | 0.11 | 1.04 |
| History of Neurological dysfunction | -16.86 | -47.17 – 13.45 | 0.275 | 1.05 |
| Previous myocardial infractions | -0.12 | -107.17 – 106.94 | 0.998 | 1.49 |
| Pre-operative dialysis | -59.81 | -122.52 – 2.90 | 0.062 | 1.08 |
| Previous percutaneous coronary intervention | 5.72 | -131.79 – 143.24 | 0.935 | 1.12 |
| History of poor mobility | -42.43 | -70.17 – -14.68 | 0.003 | 1.06 |
| Ventilated Preop | 150.31 | -3.53 – 304.16 | 0.055 | 1.25 |
| Cardiogenic Shock pre operatively | -38.92 | -130.87 – 53.03 | 0.406 | 1.28 |
| Aortic Valve Implant (Mechanical) | 59.38 | 50.89 – 67.86 | <0.001 | 1.15 |
| Cumulative Bypass time | 0.02 | -0.08 – 0.12 | 0.654 | 1.19 |
| Cumulative cross clamp time | -0.07 | -0.24 – 0.10 | 0.422 | 1.23 |
| Return to Theatre | -8.77 | -17.47 – -0.07 | 0.048 | 1.03 |
| New cerebral vascular accident | 0.04 | -16.36 – 16.44 | 0.996 | 1.04 |
| Post operative Dialysis | -55.8 | -96.26 – -15.35 | 0.007 | 1.08 |
| Patient prosthesis mismatch | 3.02 | -4.26 – 10.31 | 0.416 | 1.35 |
| Aortic valve size | -2.23 | -4.51 – 0.04 | 0.055 | 1.51 |
| Repeat valvular intervention | 19.72 | -1.79 – 41.24 | 0.072 | 1.01 |

Table S3 shows the predictors for long term survival based on pre, intra and post operative characteristics. (GVIF: Generalized Variance Inflation Factor)
